# Cost-effectiveness analysis of camrelizumab plus paclitaxel and carboplatin *versus* sintilimab plus gemcitabine and cisplatin or carboplatin for the first-line treatment of local advanced or metastatic squamous NSCLC in Chinese mainland

**DOI:** 10.3389/fphar.2024.1356725

**Published:** 2024-07-12

**Authors:** Xiaoting Liu, Xiao-xue Liu, Wenqing Shao, Yi Zhou, Jing Zhang, Cuirong Zhao, Chengwu Shen

**Affiliations:** ^1^ Department of Pharmacy, Shandong Provincial Hospital Affiliated to Shandong First Medical University, Ji’nan, Shandong, China; ^2^ Occupational Health Examination Center, Shandong Academy of Occupational Health and Occupational Medicine, Shandong First Medical University, Ji’nan, Shandong, China

**Keywords:** cost-effectiveness, squamous NSCLC, first-line treatment, camrelizumab, sintilimab

## Abstract

**Objective:**

Both camrelizumab plus paclitaxel and carboplatin (CTC) and sintilimab plus gemcitabine and cisplatin or carboplatin (SGP) have been approved by the National Medical Products Administration of China (NMPA) for the first-line treatment of local advanced or metastatic sqNSCLC. However, the comparison of the two treatments as first-line treatments in efficacy or pharmacoeconomics has barely been studied. To deeply understand the costs and outcomes of the two treatments, this work directly compared the cost-effectiveness for the first-line treatment of local advanced or metastatic squamous NSCLC in the Chinese mainland.

**Methods:**

A network meta-analysis was first performed based on the three clinical trials, namely, CameL-Sq, ORIENT-12, and C-TONG1002, to compare the clinical benefits of the two treatments. The Weibull approximation was applied to further calculate the life expectancy of the two treatments. The partitioned survival model (PSM) was next established, and one-way sensitivity analysis and probabilistic sensitivity analysis were also performed to evaluate the stability of the underlying parameter values and assumptions within the model.

**Results:**

CTC treatment gained 0.68 QALYs and cost $14,764. SGP treatment gained 0.54 QALYs and cost $14,584. The CTC arm gained 0.14 additional QALYs and cost $179 more than the SGP arm, and the ICERs was $1,269/QALY, which was lower than one-fold GDP *per capita* in the Chinese mainland ($12,734 GDP *per capita* in 2022). In probabilistic sensitivity analysis, when the WTP ranged from $12,734–38,202 (1–3 folds, 2022 GDP *per capita* in China), the CTC group had higher probabilities than the SGP group for being cost effective, which ranged from 85.65% to 88.38%.

**Conclusion:**

From the perspective of the payers, camrelizumab plus chemotherapy was cost-effective compared with sintilimab plus chemotherapy for the first-line treatment of local advanced or metastatic squamous NSCLC in the Chinese mainland.

## Introduction

Lung cancer is one of the severest threats to human health and has been reported as the second most commonly diagnosed malignancy in the world (Fitzmaurice et al., 2019). There were several new diagnosed lung cancer cases in China, which was estimated to be 828,100 in 2016 (Zheng et al., 2022). Among them, squamous non-small-cell lung cancer (sqNSCLC) accounts for approximately 25% of all kinds of lung cancer, of which major new cases are found in advanced stages or a metastatic state and barely have any known driver genes (Socinski et al., 2016). The incidence of sqNSCLC in the Chinese mainland has increased drastically in recent years, posing a significant threat to human health and placing a considerable financial burden on the Chinese healthcare system (Zhu et al., 2021). Thus, many sqNSCLC patients may neither have the chance of surgeries nor benefit from promising targeted treatments. The classic first-line treatment of local advanced or metastatic sqNSCLC was platinum-based doublet chemotherapy, from which patients only have limited benefits (Griesinger et al., 2019). Along with the study of cancer immunity and its therapeutic application, several currently available treatment options were developed in the past decade. The emergence of immune checkpoint blockers brings new hope for the treatment of local advanced or metastatic sqNSCLC, and there are several approved first-line treatments (Lahiri et al., 2023).

Camrelizumab and sintilimab are both PD-1 mono-antibody for triggering cancer immunity by the PD-1/PD-L1 checkpoint pathway. Based on the results of clinical trials, camrelizumab plus paclitaxel and carboplatin have been approved by the National Medical Products Administration of China (NMPA) for the first-line treatment of local advanced or metastatic sqNSCLC. Similarly, sintilimab plus gemcitabine and cisplatin or carboplatin were licensed by NMPA, according to the clinical trials. The two treatments were notably superior compared to platinum-based doublet chemotherapy (Zhou et al., 2021; Ren et al., 2022). In the *Guidelines of the Chinese Society of Clinical Oncology (CSCO) for Non-small-Cell Lung Cancer*, both treatments were recommended for the first-line treatment of sqNSCLC. As for second-line therapy, single treatment of docetaxel, gemcitabine, or afatinib (has not been used in first-line therapy) was recommended.

The cost-effectiveness of PD-1 antibody plus chemotherapy *versus* chemotherapy alone for the first-line treatment of NSCLC has been discussed in many research studies (Liu et al., 2022; Rui et al., 2022). Although PD-1 antibody-based immunochemotherapy shows better health outcomes in most research studies, their high price limits the cost-effectiveness when compared with chemotherapy (Shao et al., 2022). Along with decreasing the price of PD-1 antibody in China, PD-1 antibody-based immunotherapy gradually exhibited cost-effectiveness compared to chemotherapy alone. Although a sequential model has been developed to evaluate first-line and second-line treatment of sqNSCLC, which includes the two treatments as the first-line treatment (Zhao et al., 2023), a direct comparison of the two treatments in the first-line treatment of sqNSCLC in efficacy or pharmacoeconomics has barely been studied. To deeply understand the costs and outcomes of the two first-line treatment options for sqNSCLC and provide choosing advices, it is necessary to directly compare the cost-effectiveness of camrelizumab plus paclitaxel and carboplatin *versus* sintilimab plus gemcitabine and cisplatin or carboplatin for the first-line treatment of local advanced or metastatic squamous NSCLC in the Chinese mainland.

In this work, a network meta-analysis was first performed based on the three clinical trials, namely, CameL-Sq (Ren et al., 2022), ORIENT-12 (Zhou et al., 2021), and C-TONG1002 (Wang et al., 2019), to compare the clinical benefits of the two treatments. Weibull approximation was applied to further calculate the life expectancy of the two treatments (Hoyle and Henley, 2011). The partitioned survival model (PSM) was next established, and one-way sensitivity analysis and probabilistic sensitivity analysis were also performed to evaluate the stability of the underlying parameter values and assumptions within the model. Our results indicated that, from the perspective of the payers, camrelizumab plus chemotherapy was more cost-effective compared with sintilimab plus chemotherapy for the first-line treatment of local advanced or metastatic sqNSCLC. It should be noted that the comparison is between the two treatments rather than between camrelizumab and sintilimab, and the results may not reflect the difference between the two PD-1 antibodies. To the best of our knowledge, it is the first attempt to directly compare the cost-effectiveness of the two immunotherapy-based treatments, and this work may be helpful for better understanding the costs and health outcomes of the two first-line treatments of sqNSCLC in the late stages.

## Materials and methods

### Model structure

**FIGURE 1:**
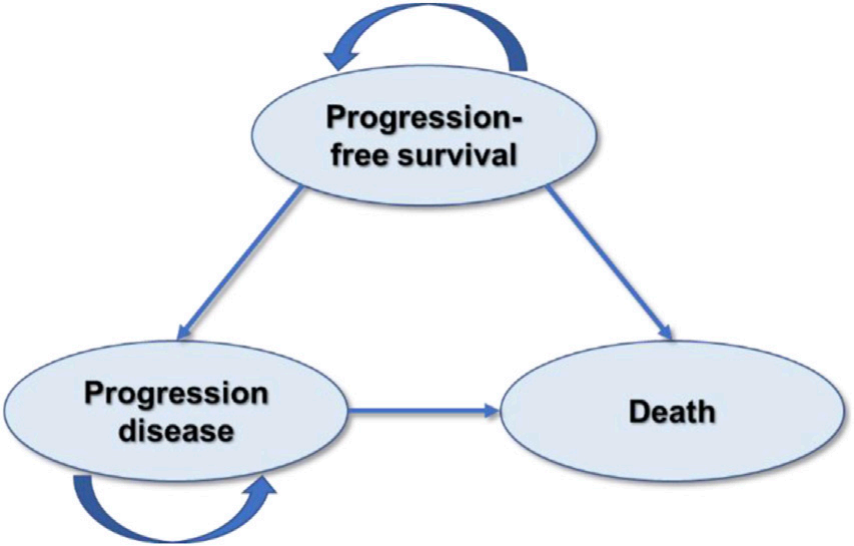
Structure of the partitioned survival model.

This study was designed and evaluated using the *Consolidated Health Economic Evaluation Reporting Standards 2022 guidelines (CHEERS)* (Husereau et al., 2022) (Supplementary Table S1). The partitioned survival model (PSM) has been chosen for cost-effectiveness analysis of camrelizumab plus paclitaxel and carboplatin *versus* sintilimab plus gemcitabine and cisplatin or carboplatin for the first-line treatment of local advanced or metastatic squamous NSCLC since it is recommended for the economic evaluation of the treatments of chronic diseases with limited health status by the *National Institute for Health and Care Excellence (NICE) DSU TECHNICAL SUPPORT DOCUMENT 19* (Woods et al., 2017) and *China Guidelines for Pharmacoeconomic Evaluations 2020* (Yue et al., 2021). The model was developed in Microsoft Excel 2021 and involved three mutually exclusive health states: progression-free survival (PFS), progression of disease (PD), and death (Figure 1). The proportion of patients in the PFS state and death state was obtained from the PFS data and overall survival (OS) data, respectively. Because there were only three states, the proportion of patients in the progression of disease state could be calculated according to that of the other two states. The cycle length was chosen as 3 weeks according to the treatment plan, and 173 cycles, in total, were conducted. The population was defined as initially diagnosed metastatic or local advanced squamous NSCLC patients eligible for the first-line treatment with CTC or SGP therapeutics.

The output data on the model were life years (LY), quality-adjusted life-years (QALYs), and cost, which were all discounted at an annual rate of 5% according to the Chinese guidelines for pharmacoeconomic evaluations. To compare the cost-effectiveness of the two arms, incremental cost-effectiveness ratios (ICERs) were calculated and presented as cost per additional LY or QALY. As the cost of new health technologies continues to rise, healthcare decision-makers across the globe are faced with the challenge of determining what constitutes a reasonable value for money. In alignment with the recommendations of the WHO-CHOICE program, the most commonly referenced CE thresholds are those based on a country’s *per capita* GDP and the estimated economic value of a year of healthy life, as determined by the Commission on Macroeconomics and Health (Bertram et al., 2016). Economists involved in the WHO-CHOICE project specifically recommend a CE threshold of less than three times the GDP *per capita* (World Health Organization, 2003). Thus, in this work, the willing-to-pay thresholds were set to be one-time to three-times the *per capita* gross domestic product (GDP) of the Chinese mainland in 2022 based on the WHO recommendations.

### Network meta-analysis

Since there were no head-to-head clinical trials of sintilimab plus GP (SGP) and camrelizumab plus TP (CTC), the network meta-analysis was first performed based on three clinical trials, namely, CameL-Sq (Ren et al., 2022), ORIENT-12 (Zhou et al., 2021), and C-TONG1002 (Wang et al., 2019), which were selected according to the *Preferred Reporting Items for Systematic Reviews and Meta-Analyses* (PRISM NMA) checklist (Supplementary Table S2). The searching strategies are illustrated in Supplementary Figure S1. The hazard ratios (HRs) of PFS and OS considered in the economic model were generated with a graph-theoretical methodology implemented in the R package netmeta (Hoyle and Henley, 2011) (Supplementary Table S3).

### Life expectancy calculation

To calculate life expectancy, GetData was chosen for data extraction (Supplementary Table S4), and Weibull approximation was used to extrapolate the PFS and OS curves of the CameL-Sq trials after comprehensive consideration of AIC (Akaike information criterion), BIC (Bayesian information criterion) (Supplementary Table S5), and visual exam of fitting, according to the literature (Billingham et al., 1999; Tappenden et al., 2006; Hoyle and Henley, 2011; Wu et al., 2014; Gu et al., 2019). The scale (λ) and shape (γ) parameters of the Weibull curves are listed in Table 1, and the survival probability at a certain time could be calculated by the formula: S(t) = exp (−λt^γ^). The Weibull fitting curve of the PFS and OS of the sintilimab plus GP arm were derived using the adjusted Weibull scale (λ_SGP_ = λ_CTC_ × HR) and shape (γ_SGP_ = γ_CTC_) parameters (Billingham et al., 1999; Tappenden et al., 2006).

**TABLE 1:** Critical parameters in the model.

Parameter	Baseline	Low	High	Distribution	Source
Survival model					
Camrelizumab PFS lambda	0.04	-	-	-	-
Camrelizumab PFS gamma	1.28	-	-	-	-
Camrelizumab OS lambda	0.01	-	-	-	-
Camrelizumab OS gamma	1.53	-	-	-	-
HR (cam/sin)					
HR_PFS	0.75	0.6	0.94	Normal	NMA
HR_OS	0.95	0.71	1.28	Normal	NMA
Discount					
Discount	0.05	0.03	0.08	-	Liu et al. (2022)
Drug costs per cycle ($)					
Camrelizumab	382.86	306.29	459.43	Gamma	Local price
Sintilimab	320.95	256.76	385.14	Gamma	Local price
Carboplatin	46.00	28.17	149.78	Gamma	Local price
Cisplatin	17.83	9.36	413.97	Gamma	Local price
Gemcitabine	50.55	38.51	728.69	Gamma	Local price
Paclitaxel	936.11	101.04	1042.47	Gamma	Local price
AE management costs ($)					
Neutrophil count decreased	115.01	51.11	357.8	Gamma	Rui et al. (2022)
White blood cell count decreased	115.01	51.11	357.8	Gamma	Rui et al. (2022)
Anemia	138.75	106.73	160.1	Gamma	Rui et al. (2022)
Platelet count decreased	1,505.92	1,240.17	1,771.67	Gamma	Rui et al. (2022)
Infectious pneumonitis	242.83	77.56	2644.56	Gamma	Jiang et al. (2021)
RCCEP	467.64	327.35	654.7	Gamma	Rui et al. (2022)
Adverse events					
CTC arm					
Neutrophil count decreased	0.55	0.44	0.66	Beta	Ren et al. (2022)
White blood cell count decreased	0.3	0.24	0.36	Beta	Ren et al. (2022)
Anemia	0.1	0.08	0.12	Beta	Ren et al. (2022)
Platelet count decreased	0.07	0.056	0.084	Beta	Ren et al. (2022)
Infectious pneumonitis	0.04	0.032	0.048	Beta	Ren et al. (2022)
RCCEP	0.11	0.088	0.132	Beta	Ren et al. (2022)
SGP arm					
Neutrophil count decreased	0.49	0.392	0.588	Beta	Zhou et al. (2021)
White blood cell count decreased	0.36	0.288	0.432	Beta	Zhou et al. (2021)
Anemia	0.34	0.272	0.408	Beta	Zhou et al. (2021)
Platelet count decreased	0.45	0.36	0.54	Beta	Zhou et al. (2021)
Infectious pneumonitis	0.14	0.112	0.168	Beta	Zhou et al. (2021)
RCCEP	0	0	0	Beta	Zhou et al. (2021)
Utility					
PFS	0.75	0.71	0.85	Beta	Rui et al. (2022)
PD	0.59	0.47	0.71	Beta	Rui et al. (2022)
Disutility					
Neutrophil count decreased	0.2	0.16	0.24	Beta	Nafees et al. (2017)
White blood cell count decreased	0.2	0.16	0.24	Beta	Nafees et al. (2017)
Anemia	0.11	0.088	0.132	Beta	Wan et al. (2019)
Platelet count decreased	0.07	0.056	0.084	Beta	Tolley et al. (2012)
Infectious pneumonitis	0.05	0.04	0.06	Beta	Zhao et al. (2023)
RCCEP	0.1	0.16	0.24	Beta	Nafees et al. (2017)

### Cost and utility data

This analysis was performed from the payers’ perspective, and only the direct medical costs were considered in the model, which included the costs of the initial treatments and maintaining treatments and the severe adverse effect (SAE) management costs. Cost data except drug costs were collected from the published articles, and the drug costs were calculated based on local price. All costs were transferred to US dollars from Chinese currency (US$ 1 = CNY ¥6.73). The utility and disutility data were also collected from the published articles.

For the SGP arm, sintilimab (200 mg), gemcitabine (1.0 g/m^2^ on day 1 and day 8 of the body surface area (BSA)), and either cisplatin (75 mg/m^2^) or carboplatin (area under the concentration–time curve (AUC) 5 mg/mL/min) were administered every 21 days for six cycles. After six cycles, patients at the PFS state continued to receive sintilimab (200 mg) monotherapy as the maintenance treatment every 21 days. Once patients entered the PD state, all treatments were suspended. Sintilimab could be administered for up to 24 months. For the CTC arm, camrelizumab (200 mg), carboplatin (AUC 5 mg/mL/min), and paclitaxel (175 mg/m^2^) were administered every 21 days for six cycles. After six cycles, patients at the PFS state continued to receive camrelizumab (200 mg) monotherapy as the maintenance treatment every 21 days. Once patients entered the PD state, all treatments were suspended. Camrelizumab could be administered for up to 24 months. The drug costs were estimated based on a typical patient who had a body surface area of 1.73 m^2^ (weight: 65 kg; height: 1.64 m) to calculate the dosage of agents according to *the Chinese Resident Nutrition and Chronic Diseases Reports 2020*. The dosage of carboplatin was calculated via the Calvert formula and the Cockcroft formula.

All SAE (incidence ≥5%, grade 3 or 4) costs were considered and are listed in Table 1. However, according to *the Chinese Clinical Use Principle of Novel Anti-cancer Drugs 2021*, grade 2–4 reactive cutaneous capillary endothelial proliferation (RCCEP), a typical AE of camrelizumab, needs special treatments. Thus, the incidence and treatment costs of grade-2 RCCEP were also involved in this model.

### Sensitivity analysis

One-way sensitivity analysis and probabilistic sensitivity analysis (PSA) were performed to evaluate the stability of the underlying parameter values and assumptions within the model. The parameters in the one-way sensitivity analysis are listed in Table 2, which were collected from literature reports and public information. Generally, the low and high limits were varied by the standard error, 95% confidence interval, or ±20% of the deterministic value.

**TABLE 2:** Baseline results of the PSM model.

	Camrelizumab plus paclitaxel and carboplatin (CTC)	Sintilimab plus gemcitabine and cisplatin or carboplatin (SGP)
LYs	1.82	1.77
PF LYs	0.89	0.72
PD LYs	0.93	1.05
QALYs	0.68	0.54
PFS QALYs	0.36	0.29
PD QALYs	0.32	0.25
Total costs	14,763.65	14,584.44
Drug costs	10,954.97	4,369.43
AE costs	3,808.68	10,215.01
Incremental costs	179.22	
Incremental LYs	0.06	
Incremental QALYs	0.14	
ICER (LYs)	3,212.01	
ICER (QALYs)	1,269.40	

As for PSA, Monte Carlo simulation was conducted via macro command in Microsoft Excel 2021. The probability distribution parameters of costs, utilities, disutility, and relative risks in the PSA were collected from published literature studies or calculated according to the mean and standard error. In this work, beta distributions were applied in the transition probability, proportion, and utilities; normal distributions were chosen for HR data from network meta-analysis; and gamma distributions were used in the costs and disutility (Rui et al., 2022). A total of 5,000 replications were performed for Monte Carlo simulation, and a set of 5,000 costs and outcomes were used to draw the cost-effectiveness scatter plot. Then, cost-effectiveness acceptability curves (CEACs) were plotted by changing the values of the willingness-to-pay threshold for an additional QALY.

## Results

### Network meta-analysis and simulation of the KM curve

**FIGURE 2:**
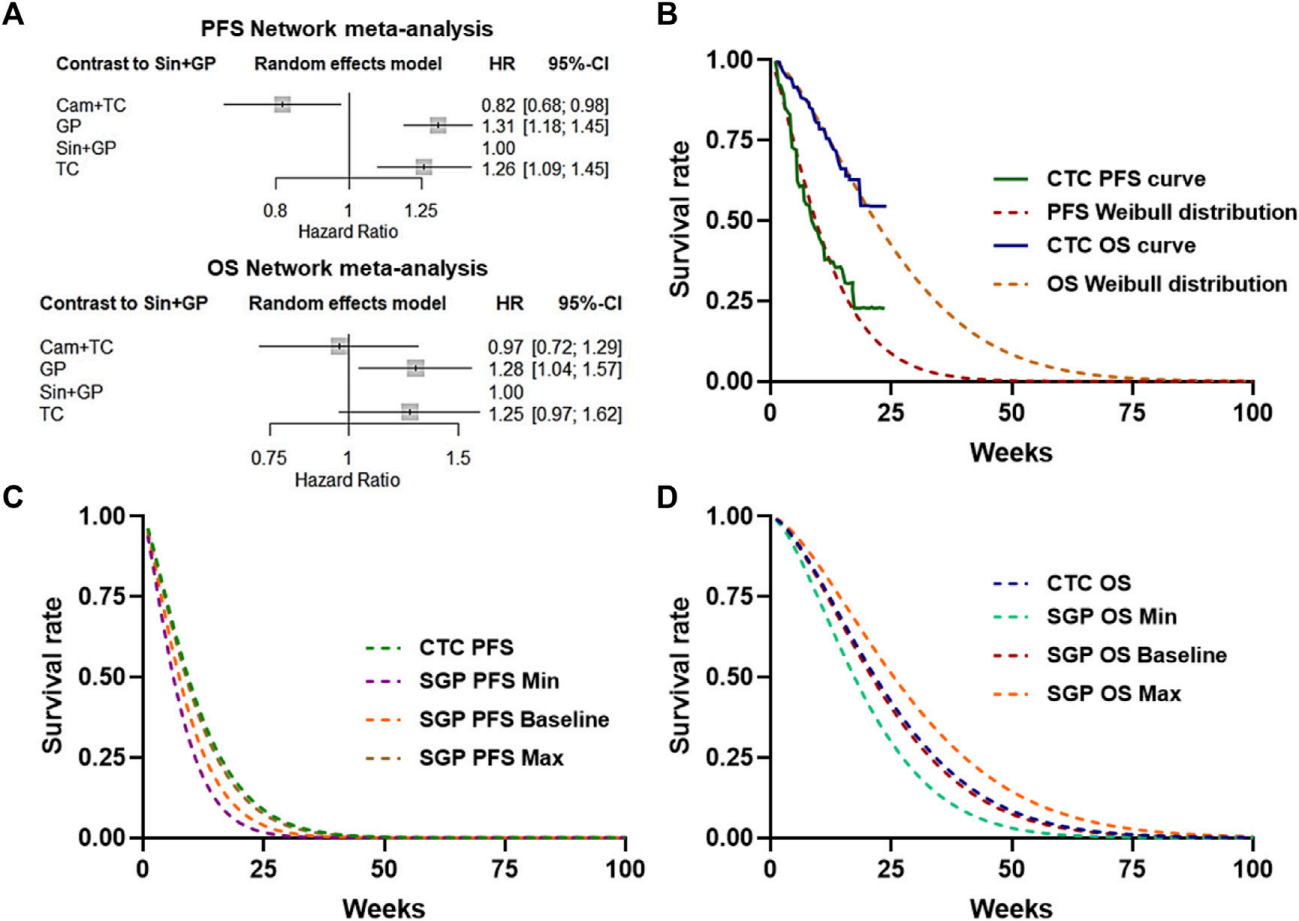
Network meta-analysis and simulation of the KM curve. **(A)** Network meta-analysis of PFS or OS of CTC, SGP, GP, and TC. **(B)** Weibull distribution of the CTC–PFS or OS–KM curve. **(C)** Baseline and 95% CI Weibull distribution of the SGP PFS survival rate. **(D)** Baseline and 95% CI Weibull distribution of the SGP OS survival rate.

Since there were no head-to-head clinical trials of sintilimab plus GP (SGP) and camrelizumab plus TP (CTC), the network meta-analysis was first performed based on three clinical trials: CameL-Sq, ORIENT-12, and C-TONG1002. The hazard ratios (HRs) of PFS and OS considered in the economic model were generated with a graph-theoretical methodology implemented in the R package netmeta (Figure 2A). To calculate life expectancy, Weibull approximation was used to extrapolate the PFS and OS curves of CameL-Sq trials according to the literature. The scale (λ) and shape (γ) parameters of the Weibull curves are listed in Table 1, and the survival probability at a certain time could be calculated by the formula: S(t) = exp (−λt^γ^). The Weibull fitting curve of the PFS and OS of the sintilimab plus GP arm were derived using the adjusted Weibull scale (λ_SGP_ = λ_CTC_ × HR) and shape (γ_SGP_ = γ_CTC_) parameters (Figures 2B–D).

### Baseline results

As listed in Table 2, the costs and health outcomes of the two arms were calculated. CTC treatment gained 0.68 QALYs and cost $14,764. SGP treatment gained 0.54 QALYs and cost $14,584. Drug costs of the CTC arm were significantly higher than those of the SGP arm, but AE costs of the CTC arm were lower than those of the SGP arm. The CTC arm gained 0.14 additional QALYs and cost $179 more than the SGP arm. Consequently, the ICER was $1,269/QALY, which was lower than one-fold GDP *per capita* in the Chinese mainland ($12,734 GDP *per capita* in 2022). Since the WTP ranged from $12,734 to 38,202 (1–3 folds 2022 GDP *per capita* in the Chinese mainland), camrelizumab plus paclitaxel and carboplatin is more cost-effective than sintilimab plus gemcitabine and cisplatin or carboplatin for the first-line treatment of local advanced or metastatic squamous NSCLC.

### Deterministic sensitivity analysis

**FIGURE 3:**
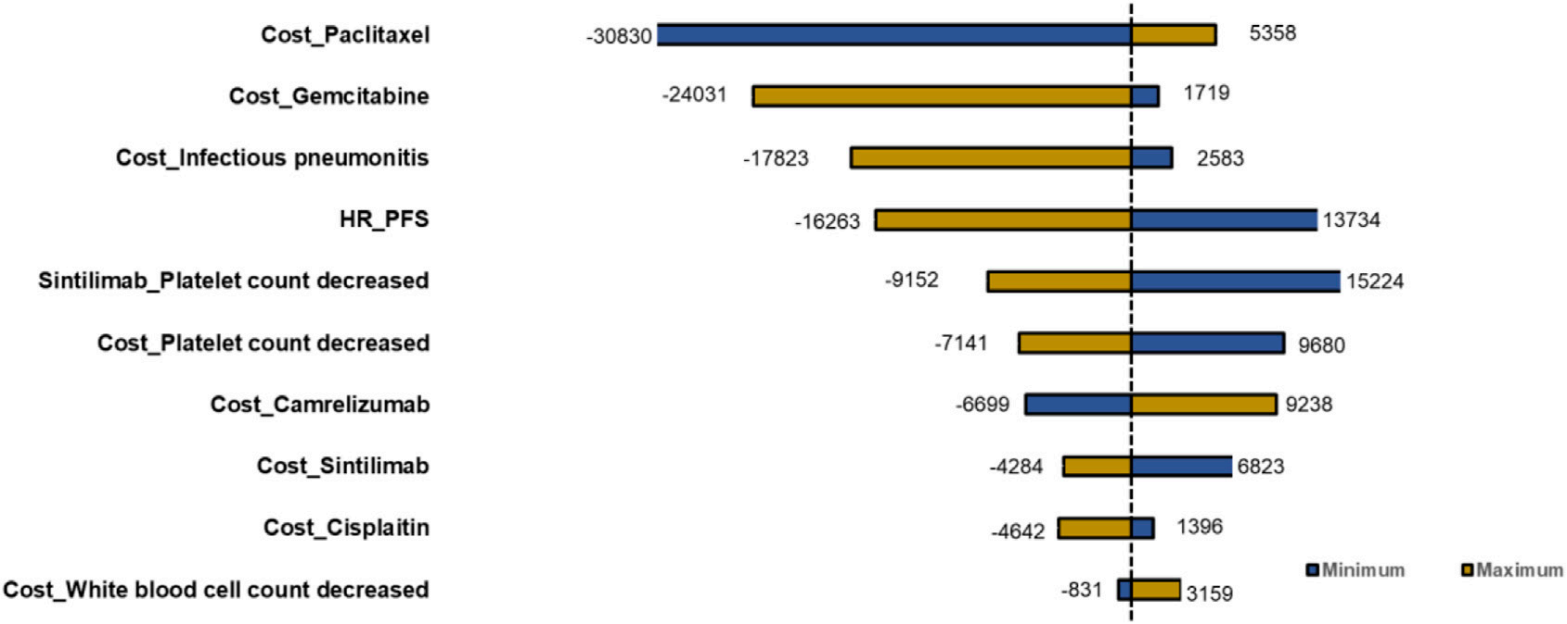
Tornado diagram of deterministic sensitivity analysis.

Deterministic sensitivity analysis was performed, and the top 10 parameters impacting ICERs are illustrated as tornado diagrams in Figure 3. The cost of paclitaxel, the cost of gemcitabine, the cost of infectious pneumonitis, the HR of the PFS curve, the cost of platelet count decreased, and the platelet count decrease chance of SGP were the main driving parameters in the DSA analysis. The significant price discrepancy between the original and generic versions of paclitaxel and gemcitabine made the costs of the two drugs the top two parameters. Specifically, gemcitabine injection (200 mg) costs $244 for the original brand and $2.9 for the cheapest generic product, while paclitaxel (30 mg) costs $98 for the original and $11 for the cheapest generic product. When the platelet count decrease of SGP or the HR of the PFS curve was set to the minimum value, the ICERs for the CTC group compared with that of the SGP group were $13,734 or $15,224 and higher than the WTP. Except the two parameters, regardless of the variation in each parameter across the wide ranges, the ICERs remained less than $12,734/QALY (1×GDP *per capita* in the Chinese mainland 2022). The DSA results indicated that the robustness of the model was acceptable.

### Probabilistic sensitivity analysis

**FIGURE 4:**
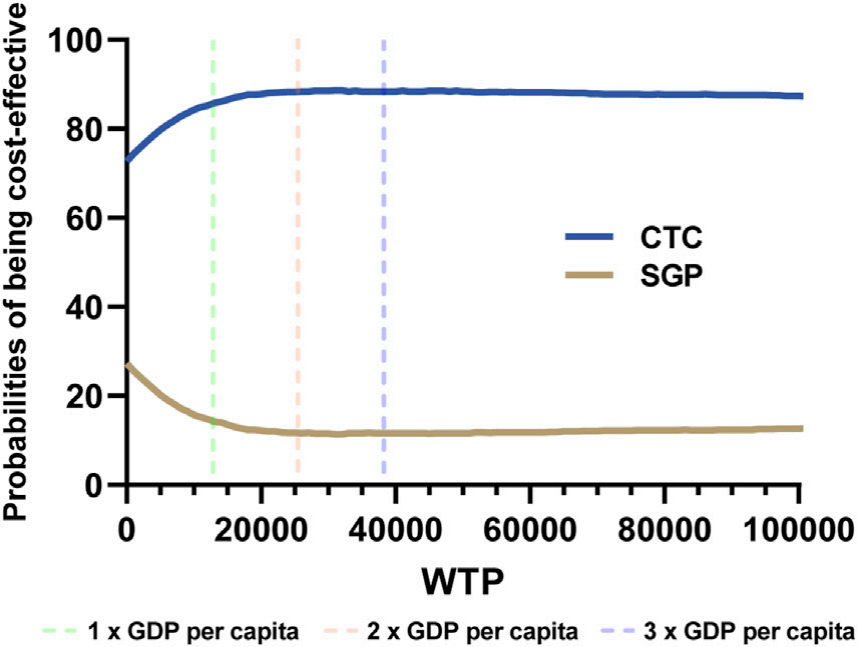
Cost-effectiveness acceptability curves of probabilistic sensitivity analysis.

In PSA analysis, 5,000 iterations of Monte Carlo simulations were carried out, and the results were summarized as cost-effectiveness acceptability curves (CECA). The average incremental QALY was 0.14, the average incremental cost was $1,972, and the probabilistic ICER was $14,086. According to Figure 4, when the WTP ranged from $12,734 to 38,202 (1–3 folds 2022 GDP *per capita* in the Chinese mainland), the CTC group had higher probabilities than the SGP group of being cost effective, which ranged from 85.65% to 88.38%.

## Discussion

Both treatment methods of camrelizumab plus paclitaxel and carboplatin and sintilimab plus gemcitabine and cisplatin or carboplatin were approved for the first-line treatment of local advanced or metastatic squamous NSCLC by the National Medical Products Administration (NMPA) of China in 2021. Since there was no head-to-head research, to compare the cost-effectiveness of the two treatments, a network meta-analysis was first performed. The base line results and CEAC indicated that camrelizumab plus paclitaxel and carboplatin was more cost-effective compared with sintilimab plus gemcitabine and cisplatin or carboplatin. To the best of our knowledge, this work was the first attempt to compare the cost-effectiveness of camrelizumab plus chemotherapy and sintilimab plus chemotherapy for the first-line treatment of local advanced or metastatic squamous NSCLC in the Chinese mainland. The results would be valuable for the Chinese payers and physicians to consider the policies and formulate a treatment plan.

Along with the development of PD-1 blocker and immunotherapy, several economic evaluations were published. Liang *et al.* reported that camrelizumab plus chemotherapy is a cost-effective first-line treatment for advanced squamous non-small-cell lung cancer in China compared with chemotherapy alone, with an ICER of $25,674 (Liang et al., 2023a). Shi *et al.* compared sintilimab combined with pemetrexed plus platinum and pemetrexed plus platinum in Chinese patients with non-squamous NSCLC who were negative for targetable genetic variations and found that the sintilimab group was cost-effective with an ICER of $5,020.74 (Shi et al., 2023). Zhang *et al.* proved that toripalimab plus chemotherapy was more cost-effective compared to chemotherapy alone for patients with advanced NSCLC in China, and the ICER was $32,237 (Zhang et al., 2023). Liang *et al.* found that tislelizumab plus chemotherapy was more cost-effective than chemotherapy alone as a first-line treatment for advanced non-squamous NSCLC in China, with an ICER of $26,162 (Liang et al., 2023b). In summary, PD-1 blockers plus chemotherapy have become the first-line therapy for various kinds of lung cancer and showed significant health outcomes and certain extra costs, but it is more cost-effective when compared to chemotherapy alone.

Another critical question is that which PD-1 blocker-based treatment is more cost-effective. Rui et al. compared camrelizumab + chemotherapy and sintilimab + chemotherapy as the first-line treatment for locally advanced or metastatic non-squamous NSCLC in China and found that sintilimab + chemotherapy had higher effectiveness (incremental QALYs ranged from 0.13–0.62) and lower total costs (incremental costs ranged from $1,099–$5,201), resulting in an ICER ranging from $6,440 to $8,454 (Rui et al., 2022). Chen *et al.* reported that sintilimab + chemotherapy appeared to be more cost-effective for the treatment of nsqNSCLC compared with sugemalimab, camrelizumab, pembrolizumab, or atezolizumab combined with chemotherapy. Chen *et al.* found that sintilimab + chemotherapy obtained more QALYs (1.2319 vs. 1.1815) and lower costs ($12,321 vs. 36,371), which implied that sintilimab + chemotherapy may dominate pembrolizumab + chemotherapy (Chen et al., 2022). These works directly compared the cost-effectiveness of different PD-1 blocker-based treatments and provided choosing advices for the clinical treatment. In this study, the direct comparison of CTC and SGP in local advanced or metastatic squamous NSCLC was performed, which supplements the gaps of previous research studies.

Our study has several strengths and limitations that should be considered. First, we used a partitioned survival model (PSM) to estimate the long-term outcomes and costs of the two chemoimmunotherapy regimens, which is a widely accepted and validated method for the economic evaluation of cancer treatments. Second, we performed a network meta-analysis (NMA) to obtain the relative efficacy of camrelizumab or sintilimab plus chemotherapy based on the data from three randomized controlled trials (RCTs) that enrolled patients with similar characteristics and used similar comparators. Third, we used reported local parameters from China to estimate the costs and utilities of the two strategies, which enhanced the applicability and relevance of our findings to the Chinese context. However, some limitations should also be acknowledged. First, the NMA had some heterogeneity and inconsistency in the pairwise comparisons, which might affect the accuracy and reliability of the indirect estimates. Although there were no direct head-to-head RCTs of the two treatments, the real-world data will be considered for further analysis of the economics and effectiveness of the two treatments. Second, we assumed that the patients received the best supportive care after disease progression, which might not reflect the actual clinical practice in China. Third, we did not include the costs and disutilities associated with grade 1/2 adverse events, which might underestimate the total costs and QALYs of the two strategies. In addition, the cost of adverse events could also be underestimated because of the emergence of new treatments for adverse events or other reasons. Therefore, further studies are needed to validate and refine our results to improve the accuracy and universality. Along with the wide application of the two treatments, multi-center real-world data would be possible for the evaluation of effectiveness and economics of CTC or SGP in future.

## Conclusion

In this study, we established a partitioned survival model (PSM) to analyze the cost-effectiveness of camrelizumab plus paclitaxel and carboplatin *versus* sintilimab plus gemcitabine and cisplatin or carboplatin for the first-line treatment of local advanced or metastatic squamous NSCLC in the Chinese mainland. From the perspective of the payers, camrelizumab plus chemotherapy was more cost-effective compared with sintilimab plus chemotherapy. However, in DSA, the risk of platelet count decrease of sintilimab and the HR of the PFS curve in network meta-analysis may impact the robustness. However, in PSA, the camrelizumab group had higher probabilities than the sintilimab group for being cost effective, which ranged from 85.65% to 88.38%, which is under 1–3 folds of the 2022 GDP *per capita* in the Chinese mainland in the CEAC, and it was consistent with our conclusion.

## Data Availability

The raw data supporting the conclusions of this article will be made available by the authors, without undue reservation.

## References

[B1] Bertram M. Y. Lauer J. A. De Joncheere K. Edejer T. Hutubessy R. Kieny M.-P. (2016). Cost-effectiveness thresholds: pros and cons. Bull. World Health Organ. 12, 925–930. 10.2471/BLT.15.164418 PMC5153921 27994285

[B2] Billingham L. J. Abrams K. R. Jones D. R. (1999). Methods for the analysis of quality-of-life and survival data in health technology assessment. Health Technol. Assess. Winch. Engl. 10, 1–152. 10.3310/hta3100 10627631

[B3] Chen P. Wang X. Zhu S. Li H. Rui M. Wang Y. (2022). Economic evaluation of sintilimab plus chemotherapy vs. pembrolizumab plus chemotherapy for the treatment of first-line advanced or metastatic squamous NSCLC. Front. Public Health 10. 10.3389/fpubh.2022.956792 PMC9395965 36016894

[B4] Fitzmaurice C. Abate D. Abbasi N. Abbastabar H. Abd-Allah F. Abdel-Rahman O. (2019). Global, regional, and national cancer incidence, mortality, years of life lost, years lived with disability, and disability-adjusted life-years for 29 cancer groups, 1990 to 2017. JAMA Oncol. 12, 2996. 10.1001/jamaoncol.2019.2996 PMC6777271 31560378

[B5] Griesinger F. Korol E. E. Kayaniyil S. Varol N. Ebner T. Goring S. M. (2019). Efficacy and safety of first-line carboplatin-versus cisplatin-based chemotherapy for non-small cell lung cancer: a meta-analysis. Lung Cancer 135, 196–204. 10.1016/j.lungcan.2019.07.010 31446995

[B6] Gu X. Zhang Q. Chu Y.-B. Zhao Y.-Y. Zhang Y.-J. Kuo D. (2019). Cost-effectiveness of afatinib, gefitinib, erlotinib and pemetrexed-based chemotherapy as first-line treatments for advanced non-small cell lung cancer in China. Lung Cancer 127, 84–89. 10.1016/j.lungcan.2018.11.029 30642557

[B7] Hoyle M. W. Henley W. (2011). Improved curve fits to summary survival data: application to economic evaluation of health technologies. BMC Med. Res. Methodol. 1, 139. 10.1186/1471-2288-11-139 PMC3198983 21985358

[B8] Husereau D. Drummond M. Augustovski F. de Bekker-Grob E. Briggs A. H. Carswell C. (2022). Consolidated Health Economic Evaluation Reporting Standards 2022 (CHEERS 2022) statement: updated reporting guidance for health economic evaluations. BMJ 376, e067975. 10.1136/bmj-2021-067975 35017145 PMC8749494

[B9] Jiang N. Li R. Bao J. Xie Y. Ma X. He Y. (2021). Incidence and disease burden of community-acquired pneumonia in southeastern China: data from integrated medical resources. Hum. Vaccin Immunother. 12, 5638–5645. 10.1080/21645515.2021.1996151 PMC8904016 34797745

[B10] Lahiri A. Maji A. Potdar P. D. Singh N. Parikh P. Bisht B. (2023). Lung cancer immunotherapy: progress, pitfalls, and promises. Mol. Cancer 22, 40. 10.1186/s12943-023-01740-y 36810079 PMC9942077

[B11] Liang X. Chen X. Li H. Li Y. (2023a). Cost-effectiveness of camrelizumab plus chemotherapy in advanced squamous non-small-cell lung cancer. Immunotherapy 15, 1133–1142. 10.2217/imt-2023-0008 37492009

[B12] Liang X. Chen X. Li H. Li Y. (2023b). Tislelizumab plus chemotherapy is more cost-effective than chemotherapy alone as first-line therapy for advanced non-squamous non-small cell lung cancer. Front. Public Health 11, 1009920. 10.3389/fpubh.2023.1009920 36794070 PMC9922748

[B13] Liu H. Wang Y. He Q. (2022). Cost-effectiveness analysis of sintilimab plus pemetrexed and platinum versus chemotherapy alone as first-line treatment in metastatic non-squamous non–small cell lung cancer in China. Health Econ. Rev. 12, 66. 10.1186/s13561-022-00410-x 36581793 PMC9801637

[B14] Nafees B. Lloyd A. J. Dewilde S. Rajan N. Lorenzo M. (2017). Health state utilities in non-small cell lung cancer: an international study. Asia Pac J. Clin. Oncol. 5, e195–e203. 10.1111/ajco.12477 26990789

[B15] Ren S. Chen J. Xu X. Jiang T. Cheng Y. Chen G. (2022). Camrelizumab plus carboplatin and paclitaxel as first-line treatment for advanced squamous NSCLC (CameL-Sq): a phase 3 trial. J. Thorac. Oncol. 4, 544–557. 10.1016/j.jtho.2021.11.018 34923163

[B16] Rui M. Fei Z. Wang Y. Zhang X. Ma A. Sun H. (2022). Cost-effectiveness analysis of sintilimab + chemotherapy versus camrelizumab + chemotherapy for the treatment of first-line locally advanced or metastatic nonsquamous NSCLC in China. J. Med. Econ. 1, 618–629. 10.1080/13696998.2022.2071066 35475459

[B17] Shao T. Ren Y. Zhao M. Tang W. (2022). Cost-effectiveness analysis of camrelizumab plus chemotherapy as first-line treatment for advanced squamous NSCLC in China. Front. Public Health 10, 912921. 10.3389/fpubh.2022.912921 36045725 PMC9423383

[B18] Shi Y. Qian D. Li Y. Chen W. Bo M. Zhang M. (2023). Comparing the cost-effectiveness of sintilimab + pemetrexed plus platinum and pemetrexed plus platinum alone as a first-line therapy for Chinese patients with nonsquamous non-small cell lung cancer. Transl. Cancer Res. 4, 928–938. 10.21037/tcr-22-2030 PMC10175008 37180653

[B19] Socinski M. A. Obasaju C. Gandara D. Hirsch F. R. Bonomi P. Bunn P. (2016). Clinicopathologic features of advanced squamous NSCLC. J. Thorac. Oncol. 9, 1411–1422. 10.1016/j.jtho.2016.05.024 27296106

[B20] Tappenden P. Chilcott J. Ward S. Eggington S. Hind D. Hummel S. (2006). Methodological issues in the economic analysis of cancer treatments. Eur. J. cancer (Oxford, Engl. 1990) 42 (17), 2867–2875. 10.1016/j.ejca.2006.08.010 17023160

[B21] Tolley K. Goad C. Yi Y. Maroudas P. Haiderali A. Thompson G. (2012). Utility elicitation study in the UK general public for late-stage chronic lymphocytic leukaemia. Eur. J. Health Econ. 5, 749–759. 10.1007/s10198-012-0419-2 22941034

[B22] Wan X. Luo X. Tan C. Zeng X. Zhang Y. Peng L. (2019). First‐line atezolizumab in addition to bevacizumab plus chemotherapy for metastatic, nonsquamous non–small cell lung cancer: a United States–based cost‐effectiveness analysis. Cancer 20, 3526–3534. 10.1002/cncr.32368 31287562

[B23] Wang Z. Huang C. Yang J.-J. Song Y. Cheng Y. Chen G.-Y. (2019). A randomised phase II clinical trial of nab-paclitaxel and carboplatin compared with gemcitabine and carboplatin as first-line therapy in advanced squamous cell lung carcinoma (C-TONG1002). Eur. J. cancer (Oxford, Engl. 1990) 109, 183–191. 10.1016/j.ejca.2019.01.007 30739019

[B24] Woods B. Sideris E. Palmer S. Latimer N. Soares M. (2017). NICE DSU technical support document 19: partitioned survival analysis for decision modelling in health care: a critical review. Available at: https://www.sheffield.ac.uk/sites/default/files/2022-02/TSD19-Partitioned-Survival-Analysis-final-report.pdf (Accessed June 2, 2017).

[B25] World Health Organization (2003). Making choices in health: WHO guide to cost-effectiveness analysis.

[B26] Wu B. Li T. Cai J. Xu Y. Zhao G. (2014). Cost-effectiveness analysis of adjuvant chemotherapies in patients presenting with gastric cancer after D2 gastrectomy. BMC Cancer 1, 984. 10.1186/1471-2407-14-984 PMC4301844 25526802

[B27] Yue X. Li Y. Wu J. Guo J. J. (2021). Current development and practice of pharmacoeconomic evaluation guidel ines for universal health coverage in China. Value Health Reg. Issues 24, 1–5. 10.1016/j.vhri.2020.07.580 33349598

[B28] Zhang M. Xu K. Lin Y. Zhou C. Bao Y. Zhang L. (2023). Cost-effectiveness analysis of toripalimab plus chemotherapy versus chemotherapy alone for advanced non-small cell lung cancer in China. Front. Immunol. 14, 1169752. 10.3389/fimmu.2023.1169752 37313403 PMC10258326

[B29] Zhao M. Shao T. Chi Z. Tang W. (2023). Effectiveness and cost-effectiveness analysis of 11 treatment paths, s even first-line and three second-line treatments for Chinese patients with advanced wild-type squamous non-small cell lung cancer: a sequent ial model. Front. public health 11, 1051484. 10.3389/fpubh.2023.1051484 36908446 PMC9999022

[B30] Zheng R. Zhang S. Zeng H. Wang S. Sun K. Chen R. (2022). Cancer incidence and mortality in China, 2016. J. Natl. Cancer Cent. 1, 1–9. 10.1016/j.jncc.2022.02.002 PMC11256658 39035212

[B31] Zhou C. Wu L. Fan Y. Wang Z. Liu L. Chen G. (2021). Sintilimab plus platinum and gemcitabine as first-line treatment for advanced or metastatic squamous NSCLC: results from a randomized, double-blind, phase 3 trial (ORIENT-12). J. Thorac. Oncol. 9, 1501–1511. 10.1016/j.jtho.2021.04.011 34048947

[B32] Zhu D. Shi X. Nicholas S. Ma Y. He P. (2021). Estimated annual prevalence, medical service utilization and direct co sts of lung cancer in urban China. Cancer Med. 8, 2914–2923. 10.1002/cam4.3845 PMC8026923 33749141

