# Supplementary material for: Cost-effectiveness analysis of camrelizumab plus paclitaxel and carboplatin versus sintilimab plus gemcitabine and cisplatin or carboplatin for the first-line treatment of local advanced or metastatic squamous NSCLC in Chinese mainland
Source: Front Pharmacol. 2024 Jul 12;15:1356725. doi: 10.3389/fphar.2024.1356725 (PMC11272525; doi:10.3389/fphar.2024.1356725)
Supplement: Supplementary file 1 [file DataSheet1.docx]

Supplementary Material

Cost-effectiveness Analysis of Carelizumab plus Paclitaxel and Carboplatin versus Sintilimab plus Gemcitabine and Cisplatin or Carboplatin for the First-line Treatment of Local Advanced or Metastatic Squamous NSCLC in Chinese Mainland

Xiaoting Liu *^1^*, Xiao-xue Liu *^2^*, Wenqing Shao *^1^*, Yi Zhou *^1^*, Jing Zhang *^1^*, Cuirong Zhao *^1^*^, *^, Chengwu Shen *^1^*^, *^

*^1^* *Department of Pharmacy, Shandong Provincial Hospital Affiliated to Shandong First Medical University, Ji'nan, Shandong, 250021, P.R. China*

*^2^ Occupational Health Examination Center, Shandong Academy of Occupational Health and Occupational Medicine, Shandong First Medical University, Ji'nan, Shandong, 250062, P.R. China*

**Table S1** CHEERS checklist

| **Section/topic** | **Item No** | **Guidance for reporting** | **Reported in section** |
| --- | --- | --- | --- |
| **Title** | | |  |
| Title | 1 | Identify the study as an economic evaluation and specify the interventions being compared. | Title |
| **Abstract** | | |  |
| Abstract | 2 | Provide a structured summary that highlights context, key methods, results, and alternative analyses. | Abstract |
| **Introduction** | | |  |
| Background and objectives | 3 | Give the context for the study, the study question, and its practical relevance for decision making in policy or practice. | Introduction |
| **Methods** | | |  |
| Health economic analysis plan | 4 | Indicate whether a health economic analysis plan was developed and where available. | Method |
| Study population | 5 | Describe characteristics of the study population (such as age range, demographics, socioeconomic, or clinical characteristics). | Method |
| Setting and location | 6 | Provide relevant contextual information that may influence findings. | Method |
| Comparators | 7 | Describe the interventions or strategies being compared and why chosen. | Method |
| Perspective | 8 | State the perspective(s) adopted by the study and why chosen. | Method |
| Time horizon | 9 | State the time horizon for the study and why appropriate. | Method |
| Discount rate | 10 | Report the discount rate(s) and reason chosen. | Method |
| Selection of outcomes | 11 | Describe what outcomes were used as the measure(s) of benefit(s) and harm(s). | Method |
| Measurement of outcomes | 12 | Describe how outcomes used to capture benefit(s) and harm(s) were measured. | Method |
| Valuation of outcomes | 13 | Describe the population and methods used to measure and value outcomes. | Method |
| Measurement and valuation of resources and costs | 14 | Describe how costs were valued. | Method |
| Currency, price date, and conversion | 15 | Report the dates of the estimated resource quantities and unit costs, plus the currency and year of conversion. | Method |
| Rationale and description of model | 16 | If modelling is used, describe in detail and why used. Report if the model is publicly available and where it can be accessed. | Method |
| Analytics and assumptions | 17 | Describe any methods for analysing or statistically transforming data, any extrapolation methods, and approaches for validating any model used. | Method |
| Characterizing heterogeneity | 18 | Describe any methods used for estimating how the results of the study vary for subgroups. | Method |
| Characterizing distributional effects | 19 | Describe how impacts are distributed across different individuals or adjustments made to reflect priority populations. | Method |
| Characterizing uncertainty | 20 | Describe methods to characterise any sources of uncertainty in the analysis. | Method |
| Approach to engagement with patients and others affected by the study | 21 | Describe any approaches to engage patients or service recipients, the general public, communities, or stakeholders (such as clinicians or payers) in the design of the study. | Method |
| **Results** | | |  |
| Study parameters | 22 | Report all analytic inputs (such as values, ranges, references) including uncertainty or distributional assumptions. | Table 1 |
| Summary of main results | 23 | Report the mean values for the main categories of costs and outcomes of interest and summarise them in the most appropriate overall measure. | Table 2 |
| Effect of uncertainty | 24 | Describe how uncertainty about analytic judgments, inputs, or projections affect findings. Report the effect of choice of discount rate and time horizon, if applicable. | Figure 3-4 |
| Effect of engagement with patients and others affected by the study | 25 | Report on any difference patient/service recipient, general public, community, or stakeholder involvement made to the approach or findings of the study | Results |
| **Discussion** | | |  |
| Study findings, limitations, generalizability, and current knowledge | 26 | Report key findings, limitations, ethical or equity considerations not captured, and how these could affect patients, policy, or practice. | Discussion |
| **Other relevant information** | | | |
| Source of funding | 27 | Describe how the study was funded and any role of the funder in the identification, design, conduct, and reporting of the analysis | Acknowledgement |
| Conflicts of interest | 28 | Report authors conflicts of interest according to journal or International Committee of Medical Journal Editors requirements. | Acknowledgement |

**Table S2** PRISMA NMA Checklist

| **Section/Topic** | | **Item #** | **Checklist Item** | **Reported in Section** |
| --- | --- | --- | --- | --- |
| **TITLE** |  | | | |
| Title | | 1 | Identify the report as a systematic review *incorporating anetwork meta-analysis (or related form of meta-analysis).* | Title |
| **ABSTRACT** | |  |  | Abstract |
| Structured summary | | 2 | Provide a structured summary including, as applicable: |  |
|  |  |  | **Background:** main objectives |  |
|  |  |  | **Methods:** data sources; study eligibility criteria, participants, and interventions; study appraisal; and *synthesis methods, such as network meta-analysis.* |  |
|  |  |  | **Results:** number of studies and participants identified; summary estimates with corresponding confidence/credible intervals; *treatment rankings may also be discussed. Authors may choose to summarize pairwise comparisons against a chosen treatment included in their analyses for brevity.* |  |
|  |  |  | **Discussion/Conclusions:** limitations; conclusions and implications of findings. |  |
|  |  |  | **Other:** primary source of funding; systematic review registration number with registry name. |  |
| **INTRODUCTION** | | | |  |
| Rationale | | 3 | Describe the rationale for the review in the context of what isalready known, including mention of why a network meta- analysis has been conducted. | NA |
| Objectives | | 4 | Provide an explicit statement of questions being addressed, with reference to participants, interventions, comparisons, outcomes, and study design (PICOS). | Introduction |
| **METHODS** | |  |  |  |
| Protocol and registration | | 5 | Indicate whether a review protocol exists and if and where it can be accessed (e.g., Web address); and, if available, provide registration information, including registration number. | NA |
| Eligibility criteria | | 6 | Specify study characteristics (e.g., PICOS, length of follow-up) and report characteristics (e.g., years considered, language, publication status) used as criteria for eligibility, giving rationale. *Clearly describe eligible treatments included in the treatment network, and note whether any have been clustered or merged into the same node (with justification).* | Method |
| Information sources | | 7 | Describe all information sources (e.g., databases with dates of coverage, contact with study authors to identify additional studies) in the search and date last searched. | Method |
| Search | | 8 | Present full electronic search strategy for at least one database, including any limits used, such that it could be repeated. | Figure S1 |
| Study selection | | 9 | State the process for selecting studies (i.e., screening, eligibility, included in systematic review, and, if applicable, included in the meta-analysis). | Figure S1 |
| Data collection process | | 10 | Describe method of data extraction from reports (e.g., piloted forms, independently, in duplicate) and any processes for obtaining and confirming data from investigators. | Table S3 |
| Data items | | 11 | List and define all variables for which data were sought (e.g., PICOS, funding sources) and any assumptions and simplifications made. | Table S3 |
| **Geometry of the network** | | **S1** | Describe methods used to explore the geometry of the treatment network under study and potential biases related to it. This should include how the evidence base has been graphically summarized for presentation, and what characteristics were compiled and used to describe the evidence base to readers. | Figure S2 |
| Risk of bias within individual studies | | 12 | Describe methods used for assessing risk of bias of individual studies (including specification of whether this was done at the study or outcome level), and how this information is to be used in any data synthesis. | NA |
| Summary measures | | 13 | State the principal summary measures (e.g., risk ratio, difference in means). Also describe the use of additional summary measures assessed, such as treatment rankings and surface under the cumulative ranking curve (SUCRA) values, as well as modified approaches used to present summary findings from meta-analyses. | Method |
| Planned methods of analysis | | 14 | Describe the methods of handling data and combining results of studies for each network meta-analysis. This should include, but not be limited to: Handling of multi-arm trials; Selection of variance structure; Selection of prior distributions in Bayesian analyses; and assessment of model fit. | Method |
| **Assessment of Inconsistency** | | **S2** | Describe the statistical methods used to evaluate the agreement of direct and indirect evidence in the treatment network(s) studied. Describe efforts taken to address its presence when found. | Method |
| Risk of bias across studies | | 15 | Specify any assessment of risk of bias that may affect the cumulative evidence (e.g., publication bias, selective reporting within studies). | NA |
| Additional analyses | | 16 | Describe methods of additional analyses if done, indicating which were pre-specified. This may include, but not be limited to, the following: *Sensitivity or subgroup analyses; Meta-regression analyses; Alternative formulations of the treatment network; and Use of alternative prior distributions for Bayesian analyses (if applicable).* | NA |
| **RESULTS†** | |  |  |  |
| Study selection | | 17 | Give numbers of studies screened, assessed for eligibility, and included in the review, with reasons for exclusions at each stage, ideally with a flow diagram. | SI |
| **Presentation of network structure** | | **S3** | Provide a network graph of the included studies to enable visualization of the geometry of the treatment network. | Figure S2 |
| **Summary of network geometry** | | **S4** | Provide a brief overview of characteristics of the treatment network. This may include commentary on the abundance of trials and randomized patients for the different interventions and pairwise comparisons in the network, gaps of evidence in the treatment network, and potential biases reflected by the network structure. | Results |
| Study characteristics | | 18 | For each study, present characteristics for which data were extracted (e.g., study size, PICOS, follow-up period) and provide the citations. | Table S3 |
| Risk of bias within studies | | 19 | Present data on risk of bias of each study and, if available, any outcome level assessment. | NA |
| Results of individual studies | | 20 | For all outcomes considered (benefits or harms), present, for each study: 1) simple summary data for each intervention group, and 2) effect estimates and confidence intervals. *Modified approaches may be needed to deal with information from larger networks.* | NA |
| Synthesis of results | | 21 | Present results of each meta-analysis done, including confidence/credible intervals. *In larger networks, authors may focus on comparisons versus a particular comparator (e.g. placebo or standard care), with full findings presented in an appendix. League tables and forest plots may be considered to summarize pairwise comparisons.* If additional summary measures were explored (such as treatment rankings), these should also be presented. | Results |
| **Exploration for inconsistency** | | **S5** | Describe results from investigations of inconsistency. This may include such information as measures of model fit to compare consistency and inconsistency models, *P* values from statistical tests, or summary of inconsistency estimates from different parts of the treatment network. | Results |
| Risk of bias across studies | | 22 | Present results of any assessment of risk of bias across studies for the evidence base being studied. | NA |
| Results of additional analyses | | 23 | Give results of additional analyses, if done (e.g., sensitivity or subgroup analyses, meta-regression analyses*, alternative network geometries studied, alternative choice of prior distributions for Bayesian analyses,* and so forth). | NA |
| **DISCUSSION** | | | | |
| Summary of evidence | | 24 | Summarize the main findings, including the strength of evidence for each main outcome; consider their relevance to key groups (e.g., healthcare providers, users, and policy- makers). | Discussion |
| Limitations | | 25 | Discuss limitations at study and outcome level (e.g., risk of bias), and at review level (e.g., incomplete retrieval of identified research, reporting bias). *Comment on the validity of the assumptions, such as transitivity and consistency. Comment on any concerns regarding network geometry (e.g., avoidance of certain comparisons).* | Discussion |
| Conclusions | | 26 | Provide a general interpretation of the results in the context of other evidence, and implications for future research. | Conclusion |
| **FUNDING** | |  |  | Acknowledgement |
| Funding | | 27 | Describe sources of funding for the systematic review and other support (e.g., supply of data); role of funders for the systematic review. This should also include information regarding whether funding has been received from manufacturers of treatments in the network and/or whether some of the authors are content experts with professional conflicts of interest that could affect use of treatments in the network. |  |

**
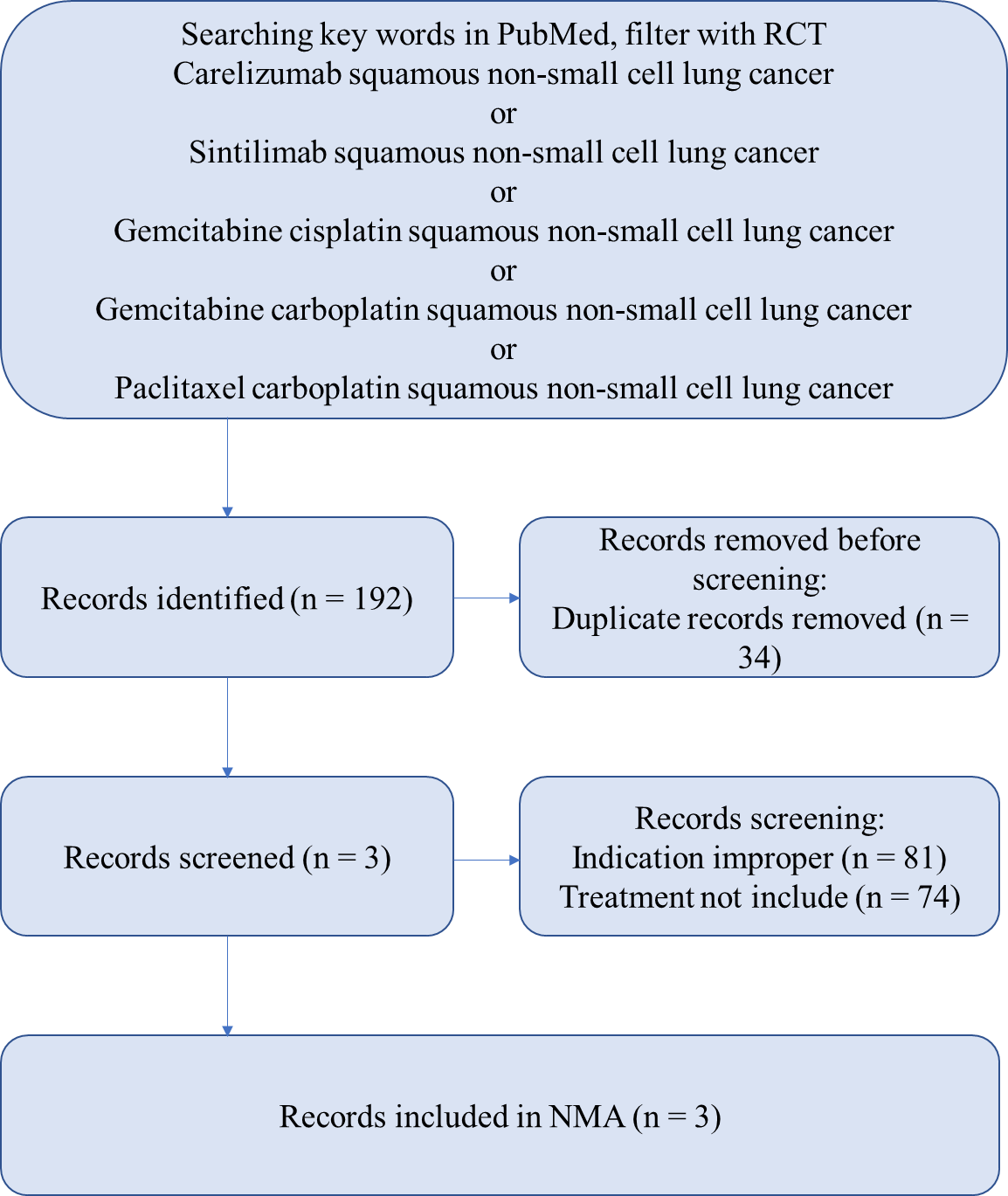
**

**Figure S1** Searching and screening strategies of researches


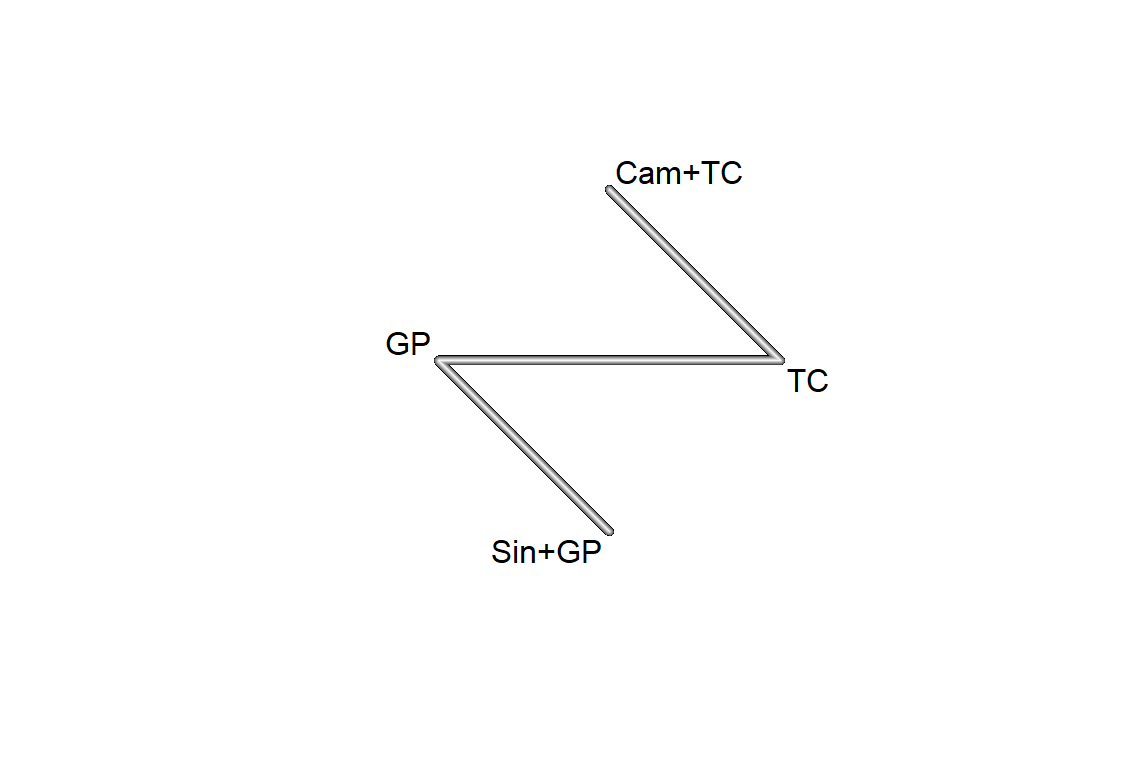


**Figure S2** the geometry of the treatment network

**Table S3** Basic characteristics of the included studies

| **Characteristic** | **CameL-Sq (N = 389)** | | **ORIENT-12 (N = 357)** | | **C-TONG1002 (N = 124)** | |
| --- | --- | --- | --- | --- | --- | --- |
|  | **Carelizumab plus GP (N = 193)** | **GP (N = 196)** | **Sintilimab plus TC (N = 179)** | **TC (N = 178)** | **GP (N = 64)** | **TC (N = 60)** |
| **Age (y)** |  |  |  |  |  |  |
| Median | 63 | 62 | 64 | 62 | 60 | 58 |
| Range | 34-74 | 34-74 | 39-75 | 33-75 | 39-76 | 41-79 |
| **Sex** |  |  |  |  |  |  |
| Male | 179 (93%) | 180 (92%) | 163 (91%) | 164 (92%) | 57 (89%) | 56 (93%) |
| Female | 14 (7%) | 16 (8%) | 16 (9%) | 14 (8%) | 7 (11%) | 4 (7%) |
| **ECOG PS** |  |  |  |  |  |  |
| 0 | 38 (20%) | 43 (22%) | 30 (17%) | 22 (12%) | 16 (25%) | 17 (28%) |
| 1 | 155 (80%) | 153 (78%) | 149 (83%) | 156 (88%) | 48 (75%) | 43 (72%) |
| **Disease stage, n (%)** |  |  |  |  |  |  |
| IIIB/IIIC | 54 (28%) | 55 (28%) | 39 (22%) | 44 (25%) | 11 (17%) | 16 (27%) |
| IV | 139 (72%) | 142 (72%) | 140 (78%) | 134 (75%) | 47 (73%) | 41 (68%) |

**Table S4** Hazard Ratios in partitioned survival model

|  | **Sin+GP/GP** | **Cam+TC/TC** | **TC/GP** |
| --- | --- | --- | --- |
| **PFS** |  |  |  |
| Baseline HR | 0.536 | 0.37 | 0.91 |
| Min HR | 0.422 | 0.29 | 0.72 |
| Max HR | 0.681 | 0.47 | 1.14 |
| **OS** |  |  |  |
| Baseline HR | 0.567 | 0.55 | 0.95 |
| Min HR | 0.353 | 0.4 | 0.67 |
| Max HR | 0.909 | 0.75 | 1.36 |

**Figure S5** CTC Survival rates data extracted from Kaplan-Meier curves

| **CTC PFS curve** | | **CTC OS curve** | |
| --- | --- | --- | --- |
| **Weeks** | **Survival rate** | **Weeks** | **Survival rate** |
| 0.968343 | 0.995924 | 1.17433 | 0.998656 |
| 1.34078 | 0.964674 | 1.24779 | 0.994624 |
| 1.65363 | 0.922554 | 1.86432 | 0.99328 |
| 2.08566 | 0.921196 | 2.01127 | 0.982527 |
| 2.68156 | 0.894022 | 2.53994 | 0.966398 |
| 2.99441 | 0.853261 | 3.40629 | 0.946237 |
| 3.6648 | 0.836957 | 3.67053 | 0.944892 |
| 4.02235 | 0.830163 | 4.03751 | 0.944892 |
| 4.17132 | 0.779891 | 4.3752 | 0.939516 |
| 4.45438 | 0.728261 | 4.80124 | 0.915323 |
| 5.28864 | 0.720109 | 5.49115 | 0.915323 |
| 5.5419 | 0.625 | 5.96111 | 0.899194 |
| 6.04842 | 0.605978 | 6.35768 | 0.883065 |
| 6.73371 | 0.610054 | 7.26785 | 0.877688 |
| 6.95717 | 0.5625 | 7.89918 | 0.86828 |
| 7.3892 | 0.550272 | 8.03151 | 0.853495 |
| 8.0298 | 0.547554 | 8.64812 | 0.846774 |
| 8.52142 | 0.5 | 9.20641 | 0.813172 |
| 9.2514 | 0.474185 | 9.51479 | 0.805108 |
| 9.75791 | 0.467391 | 9.95515 | 0.805108 |
| 10.0708 | 0.447011 | 10.161 | 0.784946 |
| 10.9795 | 0.419837 | 11.1444 | 0.784946 |
| 11.2477 | 0.377717 | 11.5852 | 0.755376 |
| 11.6499 | 0.380435 | 12.0696 | 0.755376 |
| 12.0223 | 0.377717 | 12.5249 | 0.737903 |
| 12.4395 | 0.375 | 12.9215 | 0.724462 |
| 12.9907 | 0.372283 | 13.6262 | 0.715054 |
| 13.4227 | 0.35462 | 14.0523 | 0.685484 |
| 13.9441 | 0.35462 | 14.5958 | 0.66129 |
| 14.4507 | 0.357337 | 15.5793 | 0.66129 |
| 14.8529 | 0.346467 | 15.5796 | 0.639785 |
| 15.419 | 0.307065 | 16.02 | 0.639785 |
| 16.0447 | 0.304348 | 16.3429 | 0.639785 |
| 17.013 | 0.307065 | 16.3578 | 0.627688 |
| 16.9981 | 0.285326 | 17.3559 | 0.627688 |
| 17.3855 | 0.228261 | 18.0018 | 0.627688 |
| 18.0261 | 0.228261 | 18.3101 | 0.627688 |
| 18.6518 | 0.228261 | 18.6929 | 0.547043 |
| 19.1732 | 0.22962 | 19.5296 | 0.547043 |
| 19.9628 | 0.228261 | 20.1167 | 0.547043 |
| 20.5587 | 0.228261 | 20.8213 | 0.545699 |
| 20.946 | 0.230978 | 21.2324 | 0.545699 |
| 21.2439 | 0.228261 | 21.614 | 0.545699 |
| 22.0782 | 0.228261 | 22.0103 | 0.545699 |
| 22.6443 | 0.22962 | 22.5535 | 0.545699 |
| 23.0168 | 0.228261 | 23.0525 | 0.545699 |
| 23.2998 | 0.228261 | 23.8305 | 0.545699 |
| 23.5978 | 0.225543 | 24 | 0.545699 |

**Figure S6** Summary of statistical goodness-of-fit of Kaplan-Meier curves

| **Distribution** | **CTC PFS curve** | | **CTC OS curve** | |
| --- | --- | --- | --- | --- |
|  | **AIC** | **BIC** | **AIC** | **BIC** |
| Exponential | 1074.928 | 1078.181 | 749.6886 | 752.9667 |
| Weibull | 1068.728 | 1071.98 | 739.8949 | 746.4511 |
| Logistic | 1148.053 | 1154.557 | 764.5133 | 771.0695 |
| Lognormal | 1056.304 | 1062.808 | 739.3735 | 745.9297 |
| Loglogistic | 1059.454 | 1065.959 | 739.5544 | 746.1107 |
